# How well are Brazilian mastologists (breast surgeons) trained in breast reconstruction and oncoplastic surgery? A study of the impact of a breast reconstruction and oncoplastic surgery improvement course

**DOI:** 10.3389/fonc.2023.1139461

**Published:** 2023-05-23

**Authors:** Thais Businaro Fernandes João, Vilmar Marques de Oliveira, Fábio Bagnoli, Maria Carolina Soliani Bastos, José Francisco Rinaldi, Fabrício Palermo Brenelli, Evandro Fallaci Mateus

**Affiliations:** ^1^ Irmandade da Santa Casa de Misericórdia de São Paulo (ISCMSP); Faculty of Medical Sciences of Santa Casa de São Paulo (FCMSCSP), São Paulo, Brazil; ^2^ State University of Campinas (UNICAMP), Campinas, Brazil; ^3^ Beneficiência Portuguesa de São Paulo, São Paulo, Brazil

**Keywords:** breast cancer, breast reconstruction, oncoplastic surgery, medical training, breast surgeon, reconstruction course, reconstructive breast surgery

## Abstract

**Introduction:**

The breasts are a female symbol, impacts self-image and self-esteem. Breast reconstructive and oncoplastic surgeries have an important role in minimizing injuries. In Brazil less than a third of public health system (SUS) users have access to immediate reconstructive surgery. The low rate of breast reconstructions has multiple causes and the deficiency in availability and surgeons’ technical qualification play a role. In 2010, the Breast Reconstruction and Oncoplastic Surgery Improvement Course was created by professors of the Mastology Department of Santa Casa de São Paulo and State University of Campinas (UNICAMP). The objectives of this study were to evaluate the impact of the techniques learned on patients’ management by the surgeons enrolled in the Course, as well as to characterize their profile.

**Methods:**

All students enrolled in the Improvement Course between 2010 and 2018 were invited to answer an online questionnaire. Students who did not agree to answer the questionnaire or answered them incompletely were excluded.

**Results:**

Total students included: 59. The mean age: 48.9 years, male (72%) with more than 5 years of Mastology practice (82.2%), from all regions of Brazil, 1.7% from the North, 33.9% from the Northeast, 44.1% from the Southeast, and 12% from the South. Most of the students considered they had little or no knowledge of breast reconstruction (74.6%) and 91,5% did not consider they had enough aptitude to perform breast reconstructions after finishing residency. After the Course, 96.6% considered themselves apt to perform such surgeries. Over 90% of the students considered the Course had impacted their practice and changed their surgical strategy view. Before the Course, 84.8% of the students stated that less than half of their patients who were operated on for breast cancer had breast reconstruction, compared to 30.5% after the Course.

**Conclusion:**

The Breast Reconstruction and Oncoplastic Surgery Improvement Course studied here positively impacted the mastologists’ management of patients. New training centers worldwide can help a lot of women with breast cancer.

## Introduction

Breasts are symbol of femininity. They impact on self-image, self-esteem, and the relationship between women with themselves and the world. For these reasons, breast conservative surgery (BCS) is preferred by patients (1, 2).. It is estimated that up to 30% of women who undergo BCS will have some residual deformity, many times difficult to correct (3).. Breast reconstruction began in 1895 with Vincent Czerny (4, 5). Since then, several surgical techniques have been developed and refined, such as myocutaneous flaps (3–15). Breast reconstructive and oncoplastic surgeries have an important role in minimizing injuries (13). Techniques that involve reconstruction of resection defects either by volume replacement or by volume displacement are adaptations of conventional methods of breast reconstruction or breast reduction and are applied to correct defects generated by oncological surgery (13).

In many countries, immediate reconstructive surgery is routinely offered to patients without contraindications (14). However, this is not a reality in Brazil. Less than a third of SUS´s (Sistema Único de Saúde - Brazil´s public health system) users have access to immediate reconstructive surgery (1, 16) even though they have the lawful right of having so (1). This low rate of breast reconstructions has multiple causes. Brazil´s population has important socioeconomic, ethnic and cultural diversity, and the deficiency in availability and surgeons’ technical qualification (1, 16), which makes quality care a challenge (17). A greater number of surgeons trained to perform breast reconstructions and breast repairs tend to increase the percentages of these types of surgeries. One of the alternatives is through training courses after medical specialization (16).

Developing countries, such as Brazil, tend to diagnose breast cancer at more advanced stages, which also makes it harder to carry out breast-conserving surgery (17–19).

In 2010, the Breast Reconstruction and Oncoplastic Surgery Improvement Course was created by professors of the Mastology Department of Santa Casa of São Paulo and one professor of State University of Campinas (UNICAMP) whose scope is precisely to spread the knowledge of surgical techniques for breast reconstruction.

## Objectives

The objectives of this study were to evaluate the impact of the surgical techniques taught in the Course and to characterize the profile of Brazilian mastologists (breast surgeons).

## Methods

The study was approved by the Ethics and Research in Human Beings Committee of the Santa Casa de Misericórdia of São Paulo (ISCMSP).

Between 2010 and 2013, the course was held at the Department of Obstetrics and Gynecology at ISCMSP. It was divided into 5 modules. Each module consisted of 4 hours of theoretical classes: Module 1: anatomy of the breast applied in surgery, pedicles and it´s different types and locoregional flaps (epigastric thoracic, lateral thoracic, Burrow); module 2: mastectomies and reconstructions with implants, types of prostheses and expanders, anterior chest wall anatomy, skin-sparing and nipple-sparing mastectomy and use of acellular dermal matrix (ADM); module 3: posterior thoracic wall anatomy, abdominal wall anatomy, autologous latissimus dorsi reconstruction with different techniques (extended, with prosthesis and fat grafted), single and bipedicled TRAM, flap autonomization; module 4: capsular contracture management, nipple-areola reconstruction, fat grafting, asymmetry correction; module 5: post-operative care and management of complications, proper use of surgical materials (suture, drains, dressings), instructions of patients after surgery, management of dehiscence and necrosis, management of exposed/infected protheses. The practical training had 16 hours of surgeries. On each module 8 patients, on average, were operated, with the majority of bilateral surgeries. The students were divided into groups for the practical part. This was carried out in the operating room, where the student, with the instructor of the course, performed the preoperative marking on the patient and the surgery. For each breast there was one professor teaching and guiding one student according to what was discussed and planned in the theoretical class.

This workload was divided into 2 days once a month. The activities started with the theoretical part and then the practical.

Between 2014 and 2018, the course was held at Hospital Beneficência Portuguesa in São Paulo, with 10 modules, in the same format. The themes were repeated in order to reinforce/sediment knowledge (e.g. module 1 classes were repeated on module 6). In this other format more classes were added in module 9: nipple-sparing mastectomy in irradiated breasts, pre and subpectoral reconstruction and nanolipografting; and module 10: discussion of clinical cases brought by colleagues (students) and discussion of scientific articles.

There were eight classes, seven of which were composed by 10 students and one of 12 students. The Course had seven professors-instructors. All mastologists with long experience in breast reconstructive surgery and its different techniques. Four of them are Ph.D, two MS and one MD.

All students were physicians with active Regional Medicine Council (CRMs), mastologists and with Specialist Title in Mastology (TEMa) by the Brazilian Society of Mastology and the Brazilian Medical Association. They underwent curriculum analysis and had preference for enrollment, those who had links with teaching hospitals, to serve as replicators of the acquired knowledge.

All students who took the course between 2010 and 2018 were invited. Sample calculation for this study was not necessary. They were contacted by email and phone calls and invited to answer an online questionnaire that had 38 fields and an average response time of 10 minutes. Students filled out an informed consent form agreeing to participate in the study. Exclusion criteria: students who did not agree to answer the questionnaire or answered them incompletely.

### Statistical analysis

Qualitative characteristics were described using absolute and relative frequencies, and the quantitative characteristics evaluated were described using mean and standard deviation (20). The performances of the procedures were described, and their frequencies compared, before and after the course, using McNemar test (20). For statistical purposes, in this study, surgeries were divided into complex and simple. The criterion used for this classification is the skill required by the surgeon to perform the procedure. In the group of complex surgeries, were allocated: skin sparing mastectomy with prosthesis, skin sparing mastectomy with expander, nipple sparing mastectomy with prosthesis, nipple sparing mastectomy with expander, TRAM and Latissimus dorsi flap. Simple surgeries were: sectorectomy with breast remodeling with superior/inferior/superior-medial/superior-lateral pedicle, round block, fat grafting, capsulotomy/capsulectomy and Nipple-Areola Complex Reconstruction.

Likelihood ratio tests were used to verify associations between certain characteristics of technical behavior and changes after the course was completed, based on the surgeons’ profiles. Mann-Whitney or Kruskal-Wallis tests were used to compare the percentages of changes in the reconstructions. The IBM-SPSS for Windows version 22.0 software was used to perform the analyses. For data tabulation, the Microsoft Excel 2010 software was used. The tests were performed with a significant level of 5% (p<0,05).

## Results

This study included 59 students.

**Table 1** contains personal characteristics and information about the students’ technical training. The mean age is, today, 48.9 years, most of them were male (72%), working in public and private settings simultaneously (71.2%) and with over 5 years of Mastology practice (82.2%). The Improvement Course enrolled students from all regions of Brazil, 1.7% from the North, 33.9% from the Northeast, 44.1% from the Southeast, and 12% from the South. Most of the students considered they had little or no knowledge of breast reconstruction (74.6%) and almost all of them did not consider they had enough aptitude to perform breast reconstructions after finishing residency (91.5%).

**Table 1:** Personal characteristics and information about the students’ technical training.

Variant	Description (N=59)
Age, average± SO	48,9 ± 8,2
Gender	
Female	16 (27,1)
Male	43 (72,9)
Regions	
North	1 (1’7)
Northeast	20 (33,9)
Southeast	26(44,1)
South	12 (20,3)
Medical Residency *I* Specialization	
General Surgery	14 (23,7)
Gynecology and Obstetrics	41 (69,5)
Others	4 (6,8)
Current sector of work	
Private	16 (27,1)
Private and public	42 (71,2)
Others	1 (1’7)
Consider your knowledge about breast reconstruction after leaving medical residency as:	
None	15 (25,4)
Very Little	29 (49,2)
Reasonable	9 (15,3)
Enough	1 (1’ 7)
Good	3 (5,1)
Very Good	2 (3, 4)
Did you finish the Medical Residency feeling capable of performing Breast Reconstruction surgeries?	
Yes	5 (8, 5)
No	54 (91,5)
End time of specialization*	
Up to 5 years	10 (17,9)
6 to 10 years	17 (30,4)
11 to 20 years	17 (30,4)
More than 20 anos	12 (21,4)

^*^ not all responded.

In **Table 2**, 86.4% of the students reported having as motivation to start the course the need to expand their knowledge. Before the course, 44.1% already performed reconstructions in their surgeries, but 81.4% of them were performed by a plastic surgeon. Of these mastologists, 90.6% indicated that did not perform the reconstruction because believed they did not have the necessary technical skills.

**Table 2:** Description of characteristics and opinions about the Course.

Variant	Description (N=59)
What motivated you to start the course	
Need to expand knowledge	51 (86,4)
New and reievant subject	3 (5,1)
Incentive from a mastologist colleague	5 (8,5)
Did you feel able to perfonn reconstructions surgeries aner the course?	
Yes	57 (96,6)
No	2(3,4)
In which module have you already put Into practice what you learned? *	
1st	16 (28,6)
2nd	1 (1,8)
3rd	4(7,1)
4th	8 (14,3)
5th	8 (14,3)
6th	3(5,4)
7th	5 (8,9)
8th	2 (3,6)
9th	1 (1,8)
10th	8 (14,3)
Did you already perfonn any type of reconstruction before the course?	
Yes	26 (44,1)
No	33 (55,9)
Complexity of reconstructions before the course	
Did not perform	29 (49,2)
Simple	8 (13,6)
Complex	22 (37,3)
Who perfonned the reconstructions?	
The surgeon himself	6 (10,2)
Plastic surgeon	48 (81,4)
Mastologist surgeon who already perfOrmed reconstruction	5 (8,5)
If you didn’t perfonn reconstruction before the course, why?	
Did not feel able to perform	48 (90,6)
There was no team to perfOrm	4(7,5)
I didn’think it was necessary	1 (1,9)
Complexity of reconstructions aner the course	
Simple	1 (1,7)
Complex	58 (98,3)
Quadrantectomy *I* Classic Sectorectomy	46 (78)
Did taking the course impact your daily clinical practice?	
Yes	
Changed the strategy surgical 1.1ew	54 (91,5)
I feel my patients are happier with the results.	32 (54,2)
The number of surgeries perfOrmed increased	21 (35,6)
Other impacts	3 (5,1)
Before taking the course, what Is the percentage of breast reconstructions performed In your service?	
0%	5 (8,5)
1-10%	24(40,7)
11-30”A.	13 (22)
31-50”A.	8 (13,6)
51-70”A.	1 (1,7)
*n*	5
Aner taking the course, which was the increase in the number of breast reconstructions in your service?	
!i%	1 (1,7)
1-10%	1 (1,7)
11-30”A.	3 (5,1)
31-50”A.	13 (22)
51-70”A.	15 (25,4)
71-90”A.	13 (22)
91-100%	13 (22)
Did taking the course encourage other mastologists colleagues to take the course as well?	
Yes	54 (91,5)
No	5 (8,5)
Do you keep improving/updating your knowledge in reconstruction?	
Yes	58 (98,3)
No	1 (1,7)
Types of updates	
Taking other courses	23 (39)
Congresses *I* Symposiums *I* Conierences	54 (91,5)
Literature: Books and scientific articles	42 (71,2)
Other updates	7 (11,9)
How do you rate the course in general, average ± SO	91,5 ± 13,4
How do you rate the theoretical part, average ± SO	87,4 ± 17,9
How do you rate the practical part, average± so	90,5 ± 14,4

After the Course, 96.6% considered themselves apt to perform such surgeries. After the 1st module, 28.6% put the acquired knowledge into practice. After half the course, this number reached 66.1%.

Over 90% of the students considered the Course had impacted their practice and changed their surgical strategy view. Before the Course, 84.8% of the students stated that less than half of their patients who were operated on for breast cancer had breast reconstruction, compared to 30.5% after the Course

**Tables 3**, **4** describe all the surgical techniques performed by the students, before and after completing the course. There was a statistically significant increase in the performance of all reconstruction techniques after completing the Course (p < 0.05). The muscle flap techniques (TRAM and latissimus dorsi) are the ones that students feel less confident/apt to perform.

**Table 3:** Description of the techniques used and the confidence in performing the techniques after the course.

Variant	Description (N =59)
Which types of reconstruction did you perform before the course?	
Sectorectomy with breast remodeling- superior pedicle	10 (16,9)
Sectorectomy with breast remodeling- superior- medial pedicle	7 (11,9)
Sectorectomy with breast remodeling- superior lateral pedicle	5 (8,5)
Sectorectomy with breast remodeling- inferior pedicle	4 (6,8)
Skin-sparing mastectomy with prosthesis/implant	12 (20,3)
Skin-sparing mastectomy with expander	9 (15,3)
Nipple-sparing mastectomy with prosthesis	8 (13,6)
Nipple-sparing mastectomy with expander	6 (10,2)
Round Block	19 (32,2)
TRAM	3 (5,1)
Latissimus Dorsi flap	6 (10,2)
Lipografting	2 (3,4)
Capsulectomy *I* Capsulotomy	6 (10,2)
Nipple-Areola Complex Reconstruction	3 (5,1)
After completing the course, which surgeries do you perform?	
Modified radical mastectomy without reconstruction	45 (76,3)
Sectorectomy with breast remodeling- superior pedicle	57 (96,6)
Sectorectomy with breast remodeling- superior- medial pedicle	56 (94,9)
Sectorectomy with breast remodeling- superior lateral pedicle	53 (89,8)
Sectorectomy with breast remodeling- inferior pedicle	56 (94,9)
Skin-sparing mastectomy with prosthesis/implant	56 (94,9)
Skin-sparing mastectomy with expander	53 (89,8)
Nipple-sparing mastectomy with prosthesis	58 (98, 3)
Nipple-sparing mastectomy with expander	52 (88,1)
Round Block	58 (98, 3)
TRAM	11 (18,6)
Latissimus Dorsi flap	33 (55, 9)
Lipografting	25 (42, 4)
Capsulectomy *I* Capsulotomy	50 (84, 7)
Nipple-Areola Complex Reconstruction	39 (66,1)
Which technique do you feel most confident and able to perform?	
Classic Quadrantectomy *I* Sectorectomy	31 (52, 5)
Modified radical mastectomy without reconstruction	32 (54,2)
Sectorectomy with breast remodeling- superior pedicle	48 (81, 4)
Sectorectomy with breast remodeling- superior- medial pedicle	41 (69, 5)
Sectorectomy with breast remodeling- superior lateral pedicle	36 (61)
Sectorectomy with breast remodeling- inferior pedicle	46 (78)
Skin-sparing mastectomy with prosthesis/implant	44 (74, 6)
Skin-sparing mastectomy with expander	39 (66,1)
Nipple-sparing mastectomy with prosthesis	43 (72, 9)
Nipple-sparing mastectomy with expander	37 (62, 7)
Round Block	41 (69,5)
TRAM	6 (10,2)
Latissimus Dorsi flap	23 (39)
Lipografting	15 (25, 4)
Capsulectomy *I* Capsulotomy	30 (50, 8)
Nipple-Areola Complex Reconstruction	30 (50, 8)

**Table 4:** Description of techniques performed before and after the Course and results of comparative tests.

Types of reconstruction performed	Before	After	p
Sectorectomy with breast remodeling - superior pedicle	10 (16,9)	57 (96,6)	<0,001
Sectorectomy with breast remodeling - superior - medial pedicle	7 (11,9)	56 (94,9)	<0,001
Sectorectomy with breast remodeling - superior lateral pedicle	5 (8,5)	53 (89,8)	<0,001
Sectorectomy with breast remodeling - inferior pedicle	4 (6,8)	56 (94,9)	<0,001
Skin-sparing mastectomy with prosthesis/implant	12 (20,3)	56 (94,9)	<0,001
Skin-sparing mastectomy with expander	9 (15,3)	53 (89,8)	<0,001
Nipple-sparing mastectomy with prosthesis	8 (13,6)	58 (98,3)	<0,001
Nipple-sparing mastectomy with expander	6 (10,2)	52 (88,1)	<0,001
Round Block	19 (32,2)	58 (98,3)	<0,001
TRAM	3 (5,1)	11 (18,6)	0,008
Latissimus Dorsi flap	6 (10,2)	33 (55,9)	<0,001
Lipografting	2 (3,4)	25 (42,4)	<0,001
Capsulectomy/Capsulotomy	6 (10,2)	50 (84,7)	<0,001
Nipple-Areola Complex Reconstruction	3 (5,1)	39 (66,1)	<0,001
McNemar Test			

According to **Table 5**, male professionals performed more breast reconstruction surgery and with more complex techniques before the course (p < 0.001). Despite this, there was no statistically significant difference between the sexes regarding the gain in reconstructions in the services. (p = 0.916).

**Table 5:** Description of the complexities of the techniques performed before the Course and the change in the number of procedures in the service according to gender and results of statistical tests.

Variant	Gender		Total	p
	Female	Male		
Complexity before the course				<0,001#
Did not perform	14 (87,5)	15 (34,9)	29 (49,2)	
Simple	0 (0)	8 (18,6)	8 (13,6)	
Complex	2 (12,5)	20 (46,5)	22 (37,3)	
After the course, w hat is the increase in reconstruction rates in your service?				0,916*
0%	1 (6,3)	0 (0)	1 (1,7)	
1-10%	1 (6,3)	0 (0)	1 (1,7)	
11-30%	0 (0)	3 (7)	3 (5,1)	
31-50%	3 (18,8)	10 (23,3)	13 (22)	
51-70%	5 (31,3)	10 (23,3)	15 (25,4)	
71-90%	1 (6,3)	12 (27,9)	13 (22)	
91-100%	5 (31,3)	8 (18,6)	13 (22)	
Total	16 (100)	43 (100)	59 (100)	

#Likelihood ratio test; *Mann-Whitney Test.

Finally, after analyzing the information in **Tables 6**–**8**, it is noted that the gains of students in relation to the complexity of surgeries performed before and after the Course, having as parameters the places of performance (private or private and public service), the region of activity and the time of specialization were statistically similar (p> 0.05).

**Table 6:** Description of the complexities of the techniques performed before the Course and the change in the number of procedures in the service according to the type of service and results of statistical tests.

Variant	Place of performance		Total	p
	Private	Private and Public		
Complexity before the course				0,981#
Did not perform	8 (50)	21 (50)	29 (50)	
Simple	2 (12,5)	6 (14,3)	8 (13,8)	
Complex	6 (37,5)	15 (35,7)	21 (36,2)	
After the course, w hat is the increase in reconstruction rates in your service?				0,101*
0%	1 (6,3)	0 (0)	1 (1,7)	
1-10%	0 (0)	1 (2,4)	1 (1,7)	
11-30%	0 (0)	3 (7,1)	3 (5,2)	
31-50%	3 (18,8)	10 (23,8)	13 (22,4)	
51-70%	2 (12,5)	13 (31)	15 (25,9)	
71-90%	4 (25)	9 (21,4)	13 (22,4)	
91-100%	6 (37,5)	6 (14,3)	12 (20,7)	
Total	16 (100)	42 (100)	58 (100)	

#Likelihood ratio test; *Mann-Whitney Test.

**Table 7:** Description of the complexities of the techniques performed before the Course and the change in the number of procedures in the service according to the region of trainning in Brazil and results of statistical tests.

Variant	Region					Total	p
	North	Northeast		Southeast	South		
Complexity before the course							0,629#
Did not perform	1 (100)	12 (60)		11 (42,3)	5 (41,7)	29 (49,2)	
Simple	0 (0)	2 (10)		3 (11,5)	3 (25)	8 (13,6)	
Complex	0 (0)	6 (30)		12 (46,2)	4 (33,3)	22 (37,3)	
After the course, what is the increase in reconstruction rates in your service?							0,938*
0%	0 (0)	0 (0)		1 (3,8)	0 (0)	1 (1,7)	
1-10%	0 (0)	1 (5)		0 (0)	0 (0)	1 (1,7)	
11-30%	0 (0)	1 (5)		1 (3,8)	1 (8,3)	3 (5,1)	
31-50%	0 (0)	4 (20)		4 (15,4)	5 (41,7)	13 (22)	
51-70%	1 (100)	6 (30)		8 (30,8)	0 (0)	15 (25,4)	
71-90%	0 (0)	4 (20)		6 (23,1)	3 (25)	13 (22)	
91-100%	0 (0)	4 (20)		6 (23,1)	3 (25)	13 (22)	
Total	1 (100)	20 (100)		26 (100)	12 (100)	59 (100)	

#Likelihood ratio test; *Mann-Whitney Test

**Table 8:** Description of the complexities of the techniques performed before the Course and the change in the number of procedures in the service according to graduation time and results of statistical tests.

Variável	Specialization time				Total	p
	Up to 5 years	6 to 10 years	11 to 20 years	> 20 years		
Complexity before the course						0,510#
Did not perform	3 (30)	10 (58,8)	9 (52,9)	6 (50)	28 (50)	
Simple	2 (20)	1 (5,9)	1 (5,9)	3 (25)	7 (12,5)	
Complex	5 (50)	6 (35,3)	After	3 (25)	21 (37,5)	
After the course, what is the increase in reconstruction rates in your service?						0,825*
*O”k*	0 (0)	0 (0)	1 (5,9)	0 (0)	1 (1,8)	
1-10%	1 (10)	0 (0)	0 (0)	0 (0)	1 (1,8)	
11-30%	0 (0)	0 (0)	1 (5,9)	1 (8,3)	2 (3,6)	
31-50%	2 (20)	4 (23,5)	4 (23,5)	3 (25)	13 (23,2)	
51-70%	3 (30)	4 (23,5)	6 (35,3)	2 (16,7)	15 (26,8)	
71-90%	1 (10)	6 (35,3)	1 (5,9)	4 (33,3)	12 (21,4)	
91-100%	3 (30)	3 (17,6)	4 (23,5)	2 (16,7)	12 (21,4)	
Total	10 (100)	17 (100)	17 (100)	12 (100)	56 (100)	

#Likeilhood rat1o test. *Mann-Whitney Test.

## Discussion

Medicine is a dynamic science. However, it has a traditional and conservative bias, necessary for its own safety and validation as a science. Breaking paradigms is always very challenging and adopting them, especially in practice, is not always an easy task. This was what Veronesi and Fisher observed when advocating breast conserving surgery (20–29).

Breast reconstruction can be immediate or delayed. The moment in which it is performed and the technique to be used are defined by some factors, among which are the desire and choice of the patient, stage of the disease, extension of the tumor, need or not for adjuvant therapies, comorbidities and surgical ability of the physician (30, 31). The immediate reconstruction is, in fact, an integral part of the treatment of breast cancer, even recommended by the NCCN (32), with aesthetic and psychological benefits (31), besides being less costly (32).

Although the safety, benefits and importance of oncoplasty and breast reconstruction in the treatment of breast cancer have already been well established (3, 33), it is still little performed in Brazil. The socioeconomic disparity between the regions of the country is also reflected in the screening rates, in numbers of early and advanced diagnoses, and, consequently, in different treatments for the disease (1, 17). Additionally, as we verified in the results of the analysis performed in this study, training in oncoplastic and breast reconstruction is poorly disseminated among mastologists. Most of the students, 91.5%, left the mastology residency without being able to perform such surgical techniques (**Table 1**). Of the few students who had reconstructions performed in their surgeries, almost all of them were performed by a plastic surgeon (**Table 2**).

It is certain that plastic surgery was pioneer when the subject is reconstruction and will always have its space (34). However, it is verified that there is a large number of women who do not have their breasts repaired and reconstructed, and one of the explanations is the lack of professionals able to perform such procedures. The training of a greater number of medical professionals, in fact, aims to provide an increasing number of women with the chance of having more satisfactory aesthetic results in the treatment of breast cancer, which, we cannot forget, is still very stigmatized.

Breast surgery is becoming more specialized, due to the emergence of proper courses, higher demand of patients in this direction and investment in the training of surgeons. Similar scenarios are found in other countries such as Australia, France, Germany, Italy, Portugal, Spain and United States (29)

In 2020, Brazil had 2,500 mastologists (0.6% of all medical specialists) and, in 2019, 306 physicians were pursuing medical residency in mastology (35). The number of residency programs with associated breast reconstruction teaching is uncertain. Observing the results of the present study, we can assume that this amount is low, since 91.5% of the students reported leaving the residency unable to perform reconstructions (**Table 1**).

The pioneering nature of this course, initiated by the group in 2010, and which already has seven other editions, has served as a reference, including with encouragement from the Brazilian Society of Mastology, for the implementation of others in various locations and regions of the country. The model of this course in monthly modules, with theoretical and practical content, is reproduced in other cities around the country and has proven to be very efficient (16, 29). The interest in learning about oncoplastic and breast reconstruction has been growing among mastologists, from the youngest to the most experienced.

The students participating in this study are approximately 50 years old on average today (**Table 1**). This shows that oldest medical residency programs in mastology, in fact, did not have this type of teaching. Exactly for this reason, mastologists linked to teaching hospitals were preferred to take the course. They became multipliers of knowledge, causing many medical residency programs throughout the country to offer the teaching of reconstruction.

In this study, we found that the students gained a lot of knowledge regarding oncoplastic and reconstruction techniques and began to perform these types of surgeries in their respective hospitals (**Tables 5**-**8**). The results obtained by this study indicate a higher concentration of students in the course coming from the Northeast and Southeast regions, 33.9% and 44.1%, respectively (**Table 1**). This, we believe, is a mere reflection of the distribution of mastologists across the country, which, of their total, by number obtained in 2020, 21.8% are from the Northeastern region and 51.4% from the Southeastern region (35).

In the course studied here, all students had the opportunity to learn the techniques listed in **Table 3**. Despite this, most reported less confidence in performing surgeries with muscle flaps (TRAM and Latissimus dorsi), as shown in the results presented in **Table 3** as well. This result is expected, since these are more complex techniques that require a greater learning curve from the surgeon, in addition to the greater need for constancy in their performance.

In the daily practice of the mastologists, the so-called simpler techniques, such as sectorectomies with breast remodeling and its variations, are the most commonly used. In relation to these, it was noted that the gain in knowledge was quite expressive (**Table 4**), a fact that demonstrates in a more evident manner the optimal impact of the Course.

This Course was a pioneer and served as a model for others Brazilian Mastology Society courses Around the country: Brasília, Belo Horizonte, Goiânia (16) and Jaú.

During the Course the students didn´t had any kind of effective evaluation nor report of surgery done outside, we counted on the students recall and self-reportion. For future studies some bias like recall and self-report can be avoided with implementation of test and monthly report of surgery. This way we can objectively know the number of surgeries and complication each student have and evaluate his performance and knowledge.

Another point to improve is including classes about perforator flaps, free flaps and microsurgical flaps.

## Conclusion

The Breast Reconstruction and Oncoplastic Surgery Improvement Course studied here positively impacted the mastologists’ management of patients. New training centers worldwide can help a lot of women with breast cancer.

## Data availability statement

The original contributions presented in the study are included in the article/supplementary material. Further inquiries can be directed to the corresponding author.

## Author contributions

TB: main author. VO: professor and advisor. FB: professor and co-advisor. MS: co-author. JR: professor and co-author. FP: professor and co-author. EF: co-author. All authors contributed to the article and approved the submitted version.
